# Interpretation Services in a Canadian Emergency Department: How Often Are They Utilized for Patients With Limited English Proficiency?

**DOI:** 10.7759/cureus.32288

**Published:** 2022-12-07

**Authors:** Darwin Jimal, Timothy Chaplin, Melanie Walker

**Affiliations:** 1 Emergency Medicine, Queen's University, Kingston, CAN

**Keywords:** healthy equity, emergency department operations, limited english proficiency, quality improvement and patient safety, health services accessibility

## Abstract

Introduction

Patients with limited English proficiency (LEP) face barriers to communication leading to inferior health outcomes when compared with English-proficient patients. Professional interpretation services have been shown to improve healthcare outcomes for patients with LEP but are often underutilized.

Methods

We conducted a retrospective chart review of all patients who visited the Kingston Health Sciences Centre’s ED and urgent care centre between July 2015 and August 2021 and identified as having a non-English preferred language. The demographic and visit information of LEP patients who used LanguageLine (Monterey, CA) were compared to LEP patients who did not use the service. Variables were analysed using t-tests and chi-squared tests. A survey distributed to ED physicians and residents collected perspectives on the facilitators/barriers to LanguageLine use.

Results

Of the 37,500 visits from LEP patients between 2015 and 2021, 118 (0.31%) used LanguageLine. LEP patients were more likely to access LanguageLine if they were younger (p < 0.001), had a more acute Canadian Triage Acuity Scale (CTAS) score (p < 0.001), and spoke Arabic (p<0.001). All 16 staff/residents who responded to the survey (30% response rate) had at least one LEP patient in the preceding month, and 3/16 (19%) accessed LanguageLine for these patients. Further, 5/16 (31%) reported never using the service, with 4/5 (80%) unaware the service existed. Among those aware of LanguageLine, 7/12 (58%) reported the availability of an ad-hoc interpreter as a reason for not accessing the service.

Conclusion

Interpretation services are underutilized for LEP patients in the ED, with less than 1% of these patients accessing LanguageLine. Patients were more likely to access LanguageLine if they were younger, spoke Arabic, and had a more acute triage score. Most ED physicians were either unaware of or not accessing LanguageLine despite seeing LEP patients. Future work should aim to improve the use of language services and patient-centred care for LEP patients in the ED.

## Introduction

Background

Effective communication is critical in any healthcare encounter and fundamental to building a therapeutic relationship with patients [1]. However, language barriers make it difficult for patients with limited English proficiency (LEP) to communicate effectively with their healthcare provider [2]. Language barriers can be even more pronounced in the emergency department (ED), where the time-pressured and loud environment makes it difficult to aptly describe symptoms, provide informed consent for procedures, and comprehend discharge instructions [3,4]. LEP patients are also more likely to be without a family physician and, therefore, more likely to rely on the ED than English-proficient (EP) patients [5]. These barriers can result in inferior care compared to EP patients, demonstrated by increased rates of hospital admission and readmission, increased length of stay (LOS) in the ED, increased risk of adverse reactions to medications, disparities in the number of diagnostic tests provided, increased risk of being discharged without a follow-up appointment, and an overall reduction in patient satisfaction [6-9]. Since July 2015, the Kingston Health Sciences Centre (KHSC), an academic tertiary care centre, has used LanguageLine’s (Monterey, CA) telephone interpretation service, providing clinicians with 24/7 access to a professional interpreter in over 200 languages. We sought to evaluate the use of LanguageLine in LEP patients visiting KHSC’s ED and urgent care centre (UCC).

Importance

Canada has seen a rapid growth of linguistically diverse immigration in the last decade [10]. The 2021 Canadian Census reports that over 4.7 million (12.8%) of the population have LEP [11]. Access to healthcare interpreters is paramount to ensuring compliance with the principle of accessibility outlined in the Canada Health Act [12]. While centres such as the KHSC have adopted interpretation services, no studies have looked at how often they are used in a Canadian healthcare facility. Awareness surrounding the availability and underutilization of interpretation services can increase their use and improve outcomes for LEP patients.

## Materials and methods

Study design, setting, and time period

We conducted a retrospective chart review study at an academic tertiary care emergency department and UCC in Kingston, Ontario. The ED and UCC see a combined total of approximately 100,000 patients annually. All ED and UCC visits between July 1st, 2015 and August 30th, 2021 were included. This period correlates with the implementation of the LanguageLine service. The service is accessed by telephone, often the physician's personal phone at the bedside. Once the healthcare provider connects to a LanguageLine operator, they provide the organization code, service area (i.e. urgent care, emergency department, etc.), and language of interpretation required.

Eligibility criteria

Patients were considered to have LEP if their electronic medical record (EMR) indicated a non-English preferred language. Patients were excluded from the study if their EMR did not indicate a preferred language or if they visited the ED/UCC when LanguageLine invoices were unavailable during the time period under investigation. The study population was not limited by age or reason for visits.

Data collection

Patient Demographics, ED/UCC Visit Information, and LanguageLine Use

All ED visits from July 1, 2015, to August 30, 2021, were compiled into an Excel file (Microsoft Corporation, Redmond, WA) and included the following variables: age, sex, preferred language, and Canadian Triage Acuity Scale (CTAS) score. Additionally, the file contained the central registration (CR) number of the patient involved in the encounter. Those with a non-English preferred language were filtered out. We acquired monthly LanguageLine billing invoices from our accounts payable department, which contained the CR number and the time and date of the phone call. This allowed us to cross-reference all encounters with the associated phone calls made to LanguageLine.

Physician Survey

An online Qualtrics survey was distributed weekly to all KHSC ED/UCC faculty and residents over three weeks in September 2021 to better understand the facilitators/barriers to LanguageLine use for LEP patients. Specifically, the survey included two questions about the number of LEP patients physicians had cared for in the preceding month and how often they utilized LanguageLine for those patients. Additionally, the survey included two open-ended questions to assess the barriers and facilitators to using LanguageLine.

Outcomes

The primary outcome of interest was the incidence rate, defined as the percentage of encounters where LanguageLine was used for a known LEP patient. The secondary outcomes of interest were the demographic and visit factors that influenced the provision of LanguageLine for LEP patients and the reported barriers and facilitators to using interpretation services among ED physicians.

Analysis

The incidence rate was calculated by dividing the number of ED/UCC visits involving LanguageLine by the total number of ED/UCC visits involving eligible LEP patients. We conducted a bivariate analysis using t-tests and Pearson’s chi-squared tests for continuous and categorical variables, respectively. Descriptive statistics were used to compare participant characteristics and ED/UCC visit information. P-values < 0.05 were considered statistically significant (IBM SPSS Statistics v. 27, IBM Corp., Armonk, NY). In addition, we conducted an inductive thematic analysis of the qualitative survey data to identify barriers and facilitators.

Ethical considerations

This study was approved by the Queen’s University Health Sciences and Affiliated Teaching Hospitals Research Ethics Board (# 6032655).

## Results

**Figure 1:**
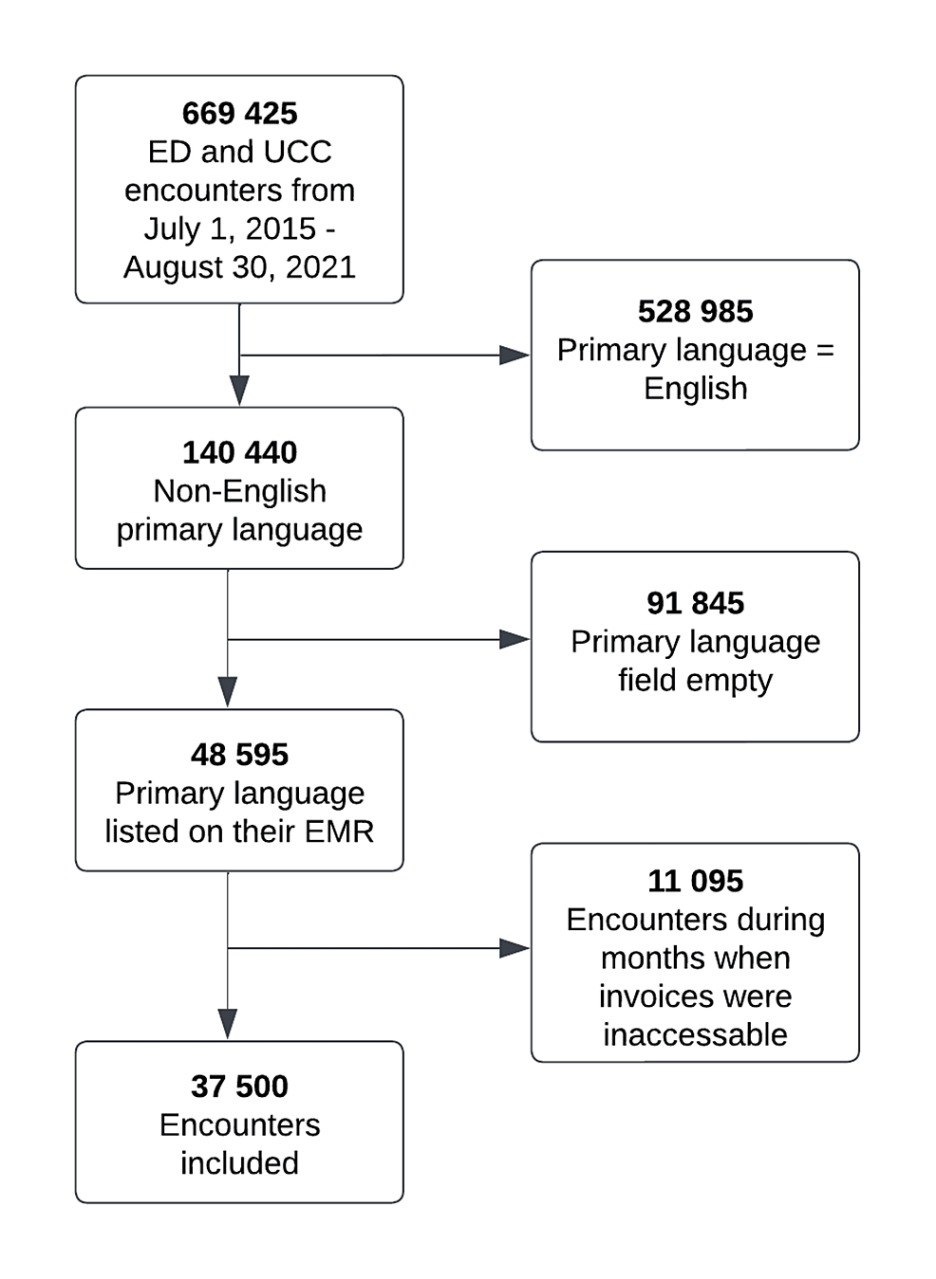
Encounter eligibility flowchart UCC: urgent care centre; EMR: electronic medical record.

As illustrated in Figure 1, nearly 670,000 encounters took place during the study period, with 37,500 meeting our eligibility criteria. LanguageLine was used for 118 (0.31%) of all eligible encounters. We excluded encounters that had an empty primary language field and those that took place during the months our organization's accounts payable department could not locate invoices. There were 64 LanguageLine calls made from the ED for encounters that had an empty primary language field.

Demographics of the study population

The patient demographics and LanguageLine use of 37,500 encounters are summarized in Table 1. Among LEP patients, those who were provided interpretation services were, on average, 12 years younger than those not offered this service (mean ± SD: 31.86 ± 21.05 and 43.42 ± 25.70, p < 0.001). LEP patients were also more likely to access LanguageLine if they had a more acute CTAS score (mean ± SD: 3.34 ± 0.98 and 3.53 ± 0.86, p < 0.001). LanguageLine was used in 18 languages, most frequently for patients who preferred Arabic (63/118 (53.4%) visits, p < 0.001). Sex was not associated with using LanguageLine.

**Table 1:** Patient demographics and LanguageLine use a. Three listed as “undifferentiated stillbirth” were excluded from the analysis. b. Categories combined due to cell size <5. c. A total of 733 patients with CTAS of 9 were excluded from the analysis. d. Other languages: Cantonese, Japanese, Finnish, Farsi, Swahili, Tigrigna, Portuguese, German, Hindi, Greek, Punjabi, and Turkish.

	LanguageLine used		LanguageLine not used		
	n (N = 118)	%	n (N = 37,382)	%	P-value
Sex					0.47
Female	60	50.8	20,239	54.1	
Male	58	49.2	17,140	45.8	
Age (years)^a^					<0.001
0-15	33	28.0	5,122	13.7	
16-44	52	44.1	14,629	39.1	
45-64	25	21.2	8,357	22.3	
≥65^b^	8	6.7	9,274	24.8	
Canadian Triage Acuity Scale (CTAS)^c^					<0.001
1-2^b^	23	18.6	4,004	10.7	
3	44	37.6	14,352	38.5	
4	35	29.9	14,068	37.7	
5	15	12.8	4,886	13.1	
Language preference					<0.001
Arabic	63	53.4	2,975	8.0	
French	8	6.8	8,788	23.5	
Mandarin	8	6.8	2,757	7.4	
Korean	7	5.9	583	1.6	
Spanish	5	4.2	2,059	5.5	
Somali	5	4.2	75	0.2	
Others^b,d^	22	18.64	20,145	53.9	

Physician survey

Our survey (Appendix) received a total of 16 responses from ED staff and residents (30% response rate). All respondents reported having at least one patient with LEP in the previous month. For those patients, only 3/16 (19%) respondents used LanguageLine. Of those who did not access LanguageLine, 5/16 (31%) reported that they had never used the service. Furthermore, 4/5 (80%) of those who had never used the service were unaware that it existed. Among those aware of LanguageLine, 7/12 (58%) reported the availability of a family member who could interpret as a deterrent to using the service.

## Discussion

We found that LanguageLine was used in less than 1% of ED visits from patients with LEP at the KHSC ED or UCC. While this study did not look at other interpretation methods, the survey responses indicate that ad-hoc interpretation from family members was common. This is concerning as ad-hoc interpretation has been shown to double the number of interpretation errors through omissions, additions, substitutions, and simplifications of what was said by both the patient and the physician [13]. In addition, patients may be uncomfortable discussing their health concerns with a family member, causing them to withhold information and delaying necessary treatment. Individuals with LEP may seek care earlier if they can reliably access interpretation services when visiting the ED.

No Canadian studies have looked at the incidence rate of interpretation services in the ED. Studies from the United States, Australia, and New Zealand demonstrate that professional interpreters are used for less than 30% of LEP patients [14]. One Australian study found that only 19.8% of LEP patients were provided professional interpretation services in the ED. Those patients were more likely to receive interpretation services if they were younger, spoke an Asian language, or used sign language [15].

The significantly low incidence rate of professional interpretation service use demonstrates a need for education and quality improvement (QI) initiatives in this area. To accommodate the needs of the growing population of Canadians with LEP, future studies should look at successful initiatives implemented at other institutions, such as introducing an “interpreter on wheels”, which significantly increased the utilization of interpretation services at one hospital in British Columbia [16]. The results of our study can serve as a starting point for other academic hospitals in Canada to examine their use of interpretation services.

Strengths and limitations

Our study has several limitations. First, our study looked at two emergency departments in Kingston, Ontario and may not represent other geographic areas with more diverse populations. Second, our accounts payable department was unable to locate the invoices for 22 months between July 2015 and August 2021. All ED visits during these months were excluded from the study. Additionally, our definition of LEP relied on a patient’s self-reported preferred language and may not be an accurate assessment of their English-speaking abilities [17]. Some LEP individuals may be overconfident in their English-speaking skills and refuse the offer of an interpreter, as English proficiency can be seen as a sign of social standing and cultural assimilation [18,19]. Finally, our data did not record other methods of interpretation that may have been used during visits, such as a family member serving as an ad-hoc interpreter, staff who could provide language-concordant care, or telephone translation applications.

Our study has several notable strengths. We reduced misclassification bias by cross-checking medical records with invoices from LanguageLine. Additionally, by incorporating a mixed-methods design, our quantitative findings were contextualized with survey results from physicians. Finally, this is one of the only studies in Canada looking at incidence rates of interpretation use and adds to the growing literature on interpretation practices in a healthcare setting.

## Conclusions

Our study found that most LEP patients who visit the KHSC ED or UCC do not utilize the hospital's interpretation service during their visit. Additionally, we found that few providers with LEP patients are accessing the service for their patients, often due to the availability of an ad-hoc interpreter, and many are unaware of its existence. This highlights the need for better education and training for healthcare providers on the importance and availability of interpretation services in the ED. Quality improvement initiatives, such as provider education and improved access to interpretation services, can help ensure more equitable access to medical care for LEP patients and reduce communication barriers.

## Appendices

Study survey

Letter of Information

Study title: The Use of Translation Services for Patients With Limited English Proficiency in the Emergency Department and Factors Associated With Its Provision.

Principle investigator: Darwin Jimal, Medical Student, Queen's University, School of Medicine.

Faculty supervisors: Dr. Melanie Walker, Queen's University, Department of Emergency Medicine and Dr. Tim Chaplin, Queen's University, Department of Emergency Medicine.

Purpose

You are invited to complete a brief survey on your use of the hospital's translation service in the last month. This survey will take one to two minutes to complete. The purpose of this survey is to gain an ED physician's perspective on how often the hospital's translation service, LanguageLine, is used for patients with limited English proficiency. It also seeks to understand what some of the barriers and facilitators are to its use.

Definition: Limited English proficiency (LEP) is a legal term referring to a level of English proficiency that is insufficient to ensure equal access to medical services without a medical interpreter. For this study, all patients with an EMR that indicates a preferred language other than English are classified as having LEP.

This study has been reviewed for ethical compliance by the Queen’s University Health Sciences and Affiliated Teaching Hospitals Research Ethics Board. There are no known risks to participating in this survey. There are no direct benefits for participating in this study. There are no penalties for not participating in this survey. Once submitted, data cannot be withdrawn as the survey is anonymous. Queen’s University Health Sciences and Affiliated Teaching Hospitals Research Ethics Board (HSREB) may require access to study-related records to monitor the ethical conduct of the research. If you have any ethics concerns, please contact the HSREB at 1-844-535-2988 or . If you have any questions about the research, please contact Darwin Jimal at .

The study data will be stored on an encrypted hard drive on KHSC servers and stored for a minimum of five years. Participant’s confidentiality will be protected to the extent permitted by applicable laws.

This Letter of Information provides you with the details to help you make an informed choice. All your questions should be answered to your satisfaction before you decide whether or not to participate in this survey.

By indicating below, I am verifying that I have read the Letter of Information, I freely consent to participate in the survey, and responses can be collected.

o Yes, you have my permission to use my responses.

Q1. In the last month, roughly how many patients with limited English proficiency have you seen in the emergency department?

o 0

o 1-3

o 4-6

o 7-9

o ≥10

Q2. How often did you access the hospital's translation service, LanguageLine, for these patients?

o Never

o Less than 10%

o 10-25%

o 26-50%

o 51-75%

o Over 75%

o Always

o I've never heard of LanguageLine

Q3. What are the reasons you do not use the hospital's translation service?

Q4. What are the reasons you use the hospital's translation service?
